# Imaging in clinical trials: a patient-led questionnaire study to assess impact of imaging regimes on patient participation

**DOI:** 10.1186/s40900-020-00195-5

**Published:** 2020-04-28

**Authors:** Katherine May, Martin Lee, Monica Jefford, Ana Ribeiro, Alison Macdonald, Veronica Morgan, Marianne Usher, Nandita M. de Souza

**Affiliations:** 1 Department of Radiology, The Royal Marsden NHS Foundation Trust, Sutton, Surrey UK; 2 Nuclear Medicine, The Royal Marsden NHS Foundation Trust, Sutton, Surrey UK; 3 NIHR Royal Marsden Clinical Research Facility, The Royal Marsden Hospital, Downs Road, Sutton, Surrey SM2 5PT UK; 4 Patient and Carer Research Review Panel, The Royal Marsden NHS Foundation Trust, Sutton, Surrey UK; 5 The Institute of Cancer Research, Sutton, Surrey UK

**Keywords:** Imaging, Clinical trials, Magnetic resonance imaging (MRI), Positron emission tomography with computerised tomography (PET/CT), Patient participation

## Abstract

**Background:**

Cancer trials often incorporate intensive imaging with Magnetic Resonance Imaging (MRI) and Positron Emission Tomography with Computerised Tomography (PET/CT), which can be physically and mentally exhausting for patients. This questionnaire study aimed to determine the aspects of imaging that affect a patient’s decision to participate in clinical trials in order to inform the design of future trials that utilise imaging. This should achieve greater patient compliance and improve the patient experience.

**Method:**

A detailed questionnaire assessing patient expectation and acceptability of imaging within clinical trials was developed in collaboration with two patient representatives. The questionnaire addressed the influence of scan type, length, frequency, scheduling, invasiveness and staff support on acceptability of imaging. It was applied to three patient groups. Group 1 consisted of patients newly recruited to studies with imaging, Group 2 consisted of previous participants in studies with imaging and Group 3 consisted of patients having imaging for clinical care.

**Results:**

One hundred ninety six patients completed the questionnaires (Group 1:47; Group 2: 50 and Group 3: 99). The use of ionising radiation and number of scans required were identified as negative influences on decision to participate by 25% of Group 3 but only by 6% of Groups 1 and 2. Scan duration >30mins was perceived as a negative factor for decision to participate by all Groups (12–22%). Good communication provided by researchers in terms of discussing the study before and after reading study materials was a key factor in influencing decision to participate (> 50% in Groups 1 and 2 and > 20% in Group 3).

**Conclusion:**

Factors relating to imaging procedures within clinical trials that affect participation have been identified with communication around study materials as the key determinant. These data will be used to influence the development of future research protocols. Modification of imaging requirements within clinical trials will improve patient tolerance and acceptability and is likely to raise recruitment.

## Plain English summary

Patients experience varying degrees of concern regarding imaging procedures; fear, claustrophobia, anxiety about radiation, fatigue, low awareness, documentation that is not clear or understandable are all considerations. There is also reluctance to attend hospital visits that are additional to scheduled treatment appointments.

This study determines what affects a patient’s decision to participate in a clinical trial involving imaging. Two experienced patient representatives were involved in the development of the patient questionnaire, specifically developed for this purpose. We assessed patient’s preferences from patients currently participating in research, patients who previously participated in research and clinical patients without previous involvement in research. The use of X-rays and numbers of scans required were negative influences on decision to participate by 25% of clinical patients but less than 10% of research patients. Scan duration >30mins was perceived as a negative factor for decision to participate by up to a fifth of all patients. Good communication by researchers in terms of discussing the study before and after reading study materials was a key factor in positively influencing decision to participate in more than half of research patients.

The results will be used to plan clinical trials involving imaging so that patient preferences are considered. This should increase future trial recruitment and patient comfort. The patient representatives will be actively involved in the dissemination of study results, within the institution, externally at conferences and through presentations to patient support groups.

## Introduction

Diagnostic medical imaging refers to techniques that allow visualisation of the structure and function of the body’s internal organs. Different imaging techniques are widely used in the study and treatment of tumours (oncology) because they are crucial for detection and staging of many cancers [1], for planning management [1–3], for assessing response to treatment [4] and for identifying recurrence of disease [5, 6]. Moreover, when introducing new imaging methods, or using refinements of existing methods, their timing in relation to treatment is also vital. Optimal scheduling of imaging ensures correct interpretation and enables different treatments such as surgery, radiotherapy and/or chemotherapy to be delivered in the most effective combination, whilst monitoring toxicity and morbidity to the patient. Imaging thus forms the mainstay of clinical research in oncology where scanning procedures of varying duration, intensity and frequency are fundamental to clinical trial protocols. Nevertheless, the perceived burden of imaging to the patient may potentially influence their decision to participate in the study as a whole. Understanding the features about the imaging itself, its scheduling and associated external factors that limit patient participation would allow imaging to be incorporated more appropriately into clinical trials and lead to greater patient acceptance.

Patient and public involvement (PPI) in research is fundamental to ensure its acceptability, relevance, timeliness, and quality. The lived experience brings added insight that can make research studies more effective [7]. Therefore, we devised a patient-led study aimed at understanding the patient perspective on participating in research studies that involved two of the main imaging methods used in cancer assessment: Magnetic Resonance Imaging (MRI) and Positron Emission Tomography with Computerised Tomography (PET/CT). MRI is a type of scan that uses strong magnetic fields and radio waves to distinguish between tissues and produce detailed images of the inside of the body. The scanner is a large tube containing powerful magnets that the patient lies inside during the scan. PET/CT scans are used to show how well certain parts of the body are working, as well as what they look like. The scanner detects the radiation given off by a radiotracer that is injected into the patient an hour before the scan. By analysing where this builds up, it is possible to identify tissues, such as cancer, that are functioning differently. The aim of this study, therefore, was to determine what factors affect a patient’s perception of MRI and PET/CT imaging and hence their decision to participate in research studies where MRI and PET/CT are included. Two patient representatives as co-investigators were engaged in the initial concept, study design, development of questionnaires, study documentation, and analysis of results.

## Methods

### Questionnaire development

A review of available validated questionnaires did not identify a suitable tool; therefore, we devised a dedicated questionnaire for the purpose of this study (Table S1). This was developed in collaboration with the two patient co-investigators and addressed imaging-specific issues and also included factors around staff support that may affect acceptability of imaging within clinical trials (GRIPP2SF, Table S2). All study documentation, questionnaires, Patient Information Sheet (PIS), consent form and invitation letter were reviewed by The Royal Marsden Patient and Carer Research Review Panel. The questionnaire development was an iterative process, involving input from a Radiologist, Research Radiographers, trial staff and patient representatives that led to a final version consisting of nine questions covering: 1) Scan parameters, including type of scan, scan preparation, scan procedure and perceived risks 2) scan scheduling, including duration and frequency 3) study participation discussion with a member of the study staff about the imaging procedures, the need for scanning and the research study and 4) external factors that could influence participation e.g. caring responsibilities, travel, costs etc. The content was based on experience of previous patient questionnaires and led by patient consultation. Testing was performed within The Royal Marsden by patient groups, radiographic and trial coordination staff. The questionnaire was individualised for the three groups.

The final versions of the questionnaire were approved by the Patient and Carer Research Review Panel at the Royal Marsden Hospital. The individual versions of the questionnaire (for groups 1, 2 and 3) are available as supplementary Table S1a, b and c.

### Patient groups

This was a single centre study conducted at The Royal Marsden Hospital over a two-year period between 2016 and 2018. Participants were patients over the age of 18 who were being treated and were due for, or had previously had, imaging in one of the following three contexts:

- **Group 1**: Currently enrolled in a research study involving imaging (MRI and/or PET/CT).
- **Group 2:** Previously enrolled in a research study that involved imaging (MRI and/or PET/CT). Patient’s involvement in the research study had ended.
- **Group 3**: Currently undergoing imaging (MRI and/or PET/CT) as part of their standard clinical care. No previous imaging as part of a research study.

Patients were eligible to participate if they met the following inclusion criteria: being treated at The Royal Marsden Hospital; over the age of 18; having imaging (MRI or PET/CT) as part of research or clinical care. Patients were excluded if they were unable to complete the questionnaire or if they had already completed a questionnaire for this study. For Group 2 patients, checks were made prior to sending out the study invitations to ensure previous participants were not deceased, to prevent causing distress. To minimise the effects of confounding variables in terms of age, gender and cancer type, recruitment was random; all eligible patients attending imaging (Groups 1 and 3)/ previously participated in imaging research studies (Group 2) who were able and willing to participate were contacted. As well as directly approaching patients, a poster advertising the study was displayed in the imaging department and questionnaires were available for patients to take to enable their participation.

Potential participants attending the MRI and PET/CT departments for scanning were approached individually by members of the study team, radiographers and trial coordinators, who administered the questionnaires. Participants were provided with a PIS explaining the rationale of the study and what their participation involved. Completed questionnaires, collected at the department reception desks, were only accepted if written consent was also provided. Patients in Group 1 completed the questionnaire after attending their first research scan. Group 2 patients were sent the information via post, along with a letter of invitation to participate and a pre-paid return envelope. Group 3 patients completed the questionnaire after their clinical scan. The study trial coordinator maintained a log of completed questionnaires.

### Data analysis

The aim of this study was to capture the factors that influence participation in trials involving imaging, namely MRI and PET/CT. Patient responses were collated from completed questionnaires and analysed by group using summary descriptive statistics. Formal testing for differences between groups was not performed, as it would not have provided additional meaningful information.

## Results

### Response to questionnaire

In total there were 196 completed questionnaires across the three groups (Group 1: 47; Group 2: 50 and Group 3: 99) with 11 responses (6%) excluded due to failure to achieve valid written consent, complete the questionnaire, or due to duplicate responses. The number of questionnaires handed to each group was not recorded, so estimation of the response rate was not possible.

### Effect of scan parameters on decision to participate

The influence of ionizing radiation on a patients’ decision to participate was greater for Group 3 patients, compared to Groups 1 and 2 patients (Table 1). Interestingly, with regards to PET/CT patients, injection of radiation affected all groups similarly. Likewise, for MRI patients, injection of a dye and acoustic noise were perceived equivalently by all groups. Lying in a tunnel was also a negative feature in Group 1 patients, but was perceived less adversely by other groups, perhaps due to less familiarity with the procedure. Overall, Group 1 and Group 2 research patients did not mind having to undergo bowel or bladder preparation procedures for scanning; this was perceived as much more of a problem by Group 3 clinical patients, particularly if pre-scan medication needed to be administered. Considering all groups together, the single most important factor negatively affecting decision to participate was use of ionising radiation.

**Table 1:** Percentage and numbers of patients undergoing imaging in current and previous studies and for clinical care only who indicate that scan preparation, scan factors, number and length of scans, scan scheduling, patient information and additional factors are a deterrent to participating in research involving imaging

Reason that would affect decision to participate	Current research patients (***n*** = 47) [%/ number of patients]	Previous research patients (***n*** = 50) [%/number of patients]	Clinical patients (***n*** = 99) [%/ number of patients]	All patients (***n*** = 196) [%/ number of patients]
**Scan preparation**				
Fasting	4.3 (2)	4.0 (2)	12.1 (12)	8.2 (16)
Bowel prep	2.1 (1)	0.0 (0)	21.2 (21)	11.2 (22)
Medication	2.1 (1)	2.0 (1)	26.3 (26)	14.3 (28)
Full bladder	2.1 (1)	6.0 (3)	13.1 (13)	8.7 (17)
**Scan factors**				
Radiation	8.5 (4)	4.0 (2)	24.2 (24)	16.3 (32)
Injecting dye	10.6 (5)	12.0 (6)	10.1 (10)	10.7 (21)
Tunnel	23.4 (11)	14.0 (7)	7.1 (7)	12.8 (25)
Injecting radiation	12.8 (6)	8.0 (4)	12.1 (12)	11.2 (22)
Noisy	14.9 (7)	14.0 (7)	12.1 (12)	13.3 (26)
**Number and length of scans**				
1–3 scans	10.6 (5)	12.0 (6)	9.1 (9)	12.2 (20)
3–5 scans	10.6 (5)	10.0 (5)	20.2 (20)	15.3 (30)
5–10 scans	4.3 (2)	10.0 (5)	38.4 (38)	23.0 (45)
Scan duration < 30 min	6.4 (3)	8.0 (4)	6.1 (6)	6.6 (13)
Scan duration < 60 min	12.8 (6)	24.0 (12)	12.1 (12)	15.3 (30)
Scan duration 60–75 min	4.3 (2)	10.0 (5)	22.2 (22)	14.8 (29)
**Scheduling of scans**				
9 am – 5 pm	14.9 (7)	12.0 (6)	19.2 (19)	16.3 (32)
Evenings and weekends	4.3 (2)	6.0 (3)	30.3 (30)	17.9 (35)
Alternative times	21.3 (10)	16 (8)	20.2 (20)	19.4 (38)
**Patient information on imaging**				
Discussion before	59.6 (28)	52.0 (26)	19.2 (19)	37.2 (73)
Discussion after PIS	40.4 (19)	30.0 (15)	19.2 (19)	27.0 (53)
**Additional factors**				
Additional visits	21.3 (10)	16.0 (8)	32.3 (32)	25.5 (50)
Flexibility	23.4 (11)	28.0 (14)	40.4 (40)	33.2 (65)
Time off work	10.6 (5)	8.0 (4)	35.4 (35)	22.4 (44)
Childcare	0.0 (0)	2.0 (1)	17.2 (17)	9.2 (18)
Carer responsibilities	0.0 (0)	6.0 (3)	13.1 (13)	8.2 (16)
Cost of travel	10.6 (5)	8.0 (4)	32.3 (32)	20.9 (41)
Travel time	19.1 (9)	8.0 (4)	45.5 (45)	29.6 (58)
Parking	14.9 (7)	20.0 (10)	45.5 (45)	31.6 (62)

### Effect of scan scheduling on decision to participate

The number of scans adversely affected participation primarily among Group 3 clinical patients: nearly 40% of these patients indicated that more than 5 scans within the study would be a deterrent to participation (Table 1). Not surprisingly, scan duration of less than 30 min appeared acceptable to 93.4% of patients from all groups, but longer scan durations became more burdensome. However, scan duration was less problematic than the number of scans, which adversely influenced the decision in twice the number of patients, particularly from Group 3. Surprisingly, Group 1 and Group 2 patients preferred the option of evening or weekend scans, Group 3 patients did not (Table 1). This may well have been influenced by ability to get time off work which may be possible in a clinical but not in a research context.

### Effect of discussion about study participation

Discussion with imaging researchers (radiographers, radiologists) about study participation and what that involved was a very large factor on decision to participate in research, both for Group 2 patients who had previously participated, and for Group 1 who were currently recruited to research (Table 1). Group 3 clinical patients perceived this factor as being less important in their decision to participate, both before and after reading the PIS.

### Effect of external factors on decision to participate

The time off work, childcare and carer responsibilities was perceived as influencing decision to participate in Group 3 (clinical) patients, but was much less important to research participants in Groups 1 and 2 (Table 1).

Travel time and cost of travel also was perceived as a deterrent by Group 3 clinical patients, but appeared to be less of a problem for Group 1 and 2 patients. Interestingly, the effect of parking was substantial, particularly for Group 3 patients (Table 1).

## Discussion

This patient-led study indicates that the key factor in affecting a patient’s decision to undergo imaging procedures as part of a research study is the support provided by the researchers on the imaging component of the study. Discussion was deemed essential not just before reading the PIS, but also after reading it, so that queries could be addressed and clarifications provided on matters arising. The latter is interesting given that a comprehensive, understandable PIS has always been at the heart of the development of any study, where input from patients is routinely sought and incorporated to make the information accessible. Despite this, reading such a document needs to be supplemented by a detailed conversation with staff on the specifics of what the study involves and in order to seek reassurance about safety, tolerability, and support throughout; the availability of this support is of primary importance to all potential recruits when deciding to participate in research. At our facility, the primary study discussion is with the study chief investigator/consultant radiologist or medically responsible deputy, who goes through the PIS with the patient as part of the informed consent process. Further discussion takes place with the research radiographer when attending for scanning. Both these interactions are important in research participation and time is factored for this into research scan scheduling. More specifically, this observation also correlates with the results of a recent study undertaken to determine radiation exposure awareness of patients undergoing diagnostic nuclear medicine scans. This highlighted both that patients are not well informed of the radiation exposure related to these imaging procedures and that they would like greater communication with healthcare professionals to provide them with the information in a meaningful way [8]. Lying in a tunnel was identified as a negative feature in nearly 25% of Group 1 patients. For a research study which involves patients having whole body MRI scans (WB-MRI), we have worked closely with a patient contributor to develop information that is reflective of the real patient experience of having a WB-MRI scan, enabling better description of the procedure to facilitate participants’ understanding. The provision of clearly explained scan information is crucial for patient acceptability and positive experience. It could also impact on recruitment, tolerance and compliance [9].

The lack of impact of scan type on decision to participate was interesting, particularly in view of the administration of radiation dose. Clinical patients (Group 3) perceived this as a problem, but research participants (Groups 1 and 2) did not. This may well be because clinical patients may have been at an earlier stage of disease, or at a stable phase in remission, where the potential harmful effects of radiation were a consideration. Patients in our oncology centre being consented for research trials involving imaging would likely have been at a later stage of disease, or where current treatments had failed, so that the perceived harmful effects of radiation were of lesser importance in this context. Injected radiation seemed also to have a greater effect on current rather than previous research participants. Again, this may be related to anxieties around long-term deposition of radioactive agents, and a lack of experience of being exposed to such agents. This further highlights the importance of good and accurate information about the technical aspects of the imaging techniques, potential morbidities and education on radiation safety. All of these require discussion with experts familiar with the technique either before or after the potential recruit has read the PIS, while deciding whether to participate. This correlates with Thornton et al. [10], who found that understanding imaging radiation risks and active participation in decision making about imaging were especially important to cancer survivors. It is noteworthy that research participants were generally more accepting and tolerant of research-associated factors than clinical patients. This could indicate positive previous experiences, removal of fear of the unknown or that previous participants are already signed up to the fundamental principles of research. Childcare and carer arrangements for extra visits were not a major issue. It is often perceived by healthcare workers and researchers that this may be a limiting factor in patient participation, but this was not the case. This could be a reflection, as a specialist oncology referral centre, of our patient demographic, where the majority of patients tend to be more elderly. This is further borne out by the fact that only a minority of patients considered travel time and costs a limiting factor. Research studies normally have invested resource in ensuring that travel for patients is simplified by budgeting for taxi transport, but this only affects a minority of patients. What appears to be more important is to ensure that patients driving to a site for additional appointments do not have the stress of being unable to park, but that there are clearly ring-fenced spaces for this purpose.

Patient input into questionnaire development through multiple iterations with final review by the hospital’s patient and carer review panel improved the quality of the final questionnaire. It made the tool clear and simple to use and identified factors that were likely to be critically important to patients (such as parking facilities) when they must attend for additional appointments. We did not feel it justifiable however, to burden patients with filling in 2 questionnaires, devised without and with patient input to assess whether the compliance or results would have been any different. Limitations include lack of depth of the questionnaire, however through consultation with patient groups, it was felt that a more detailed or lengthy survey would mean a poorer completion rate. The timing of questionnaire administration may also have introduced bias, as patients in the imaging suite may have been positively or negatively influenced by their current experience. Other sources of bias include interaction with the same radiographer for questionnaire as for the scan, and completing the questionnaire at home rather than on site. Moreover, not all patients in our cohorts would have had experience of all scan types. Some may have had brief MRI scans; some may have had more intensive investigations or had multiple scan appointments. Also, this research focused on the experiences of participants in oncology trials at a tertiary referral centre. However, it has begun to identify those factors that may adversely affect the uptake of such studies. This knowledge can now be applied more widely to the development of future research studies involving imaging, with the aim of raising recruitment and patient compliance as well as improving the research experience for the participant. Our patient representatives work within an established network [9, 11] with outreach to specialist patient cancer focus groups and the National Cancer Research Institute, which will aid dissemination of these findings and raise awareness of the role of imaging in clinical trials.

## Conclusion

This patient-led study, in current and previous imaging research participants and in patients being imaged for clinical care only, examined factors that influence a patient’s decision to participate in trials with imaging. It incorporated full patient involvement to ensure the patient perspective. The results indicate that discussion with research staff both before and after reading the PIS are perceived as key to patient recruitment into trials involving imaging, particularly by research patients. The use of ionising radiation, number of scans (> 3) or scan duration (> 60 mins), time off work, flexibility, cost and time of travel was perceived as a barrier by 20–50% of patients imaged for clinical care, but less so for those currently or previously involved with imaging research. Since participation in imaging studies encompasses a higher degree of altruism as compared to therapeutic trials, it is crucial that discussion about these studies and acceptability of the imaging regimens is ensured to maximise recruitment. This should advance the development and use of novel imaging methods for research trials and ultimately for clinical care.

## Supplementary information

**Additional file 1.**

## Data Availability

Not Applicable.

## Abbreviations

PPI: Patient and public involvement
MRI: Magnetic resonance imaging
PET/CT: Positron emission tomography with computerised tomography
PIS: Patient information sheet
WB-MRI: Whole body MRI
