# Supplementary material for: Imaging in clinical trials: a patient-led questionnaire study to assess impact of imaging regimes on patient participation
Source: Res Involv Engagem. 2020 Apr 28;6:15. doi: 10.1186/s40900-020-00195-5 (PMC7189543; doi:10.1186/s40900-020-00195-5)
Supplement: Supplementary file 1 — Additional file 1. [file 40900_2020_195_MOESM1_ESM.zip › Table 1a_Questionnaire - Group 1 - V4 - 07-04-15R1.docx]

**Imaging Study Questionnaire**

**Group 1 – Patients Currently Involved in Research**

This questionnaire is aimed at finding out what influences patients to participate in research studies that involve having imaging scans, in particular MRI and PET/CT. We are inviting people to answer the questionnaire who have experience of participating in imaging research studies as well as those who have only clinical experiences with imaging.

1. You are currently participating in a research study using imaging? What imaging is involved?

PET MRI CT Other

How many scans are involved?

1 2-3 more than 3

1. Was your decision to participate in the study affected by the scheduling involved:

Yes No Unsure

- 1. Additional visits to the hospital
  2. Flexibility in day/time of scan
  3. Time off work
  4. Childcare responsibilities at home
  5. Carer responsibilities at home
  6. Cost of travel to/from the hospital
  7. Travel time to/from the hospital
  8. Parking availability
  9. Discussion with member of staff about the study
     **before** reading the patient information sheet
  10. Discussion with member of staff about the study
      **after** reading the patient information sheet

1. Was your decision to participate in the study affected by the scan preparation involved:

Yes No Unsure

1. Fasting beforehand for up to 4 hours
2. Bowel preparation
3. Additional medication
4. Having a full bladder
5. Was your decision to participate in the study affected by the scan involved:

Yes No Unsure

1. Radiation
2. Injection of a dye
3. Lying in a short tunnel
4. Injection of a short-lived radioactive substance
5. Excessive noise
6. Was your decision to participate in the study affected by the length of the scan involved:

Yes No Unsure

- 1. Less than 30 minutes
  2. Less than 60 minutes
  3. 60-75 minutes

1. Was your decision to participate in the study affected by the number of scans required over
   a 12-month period: Yes No Unsure
2. 1-3 scans
3. 3-5 scans
4. 5-10 scans
5. Was your decision to participate in the study affected by your appointment timing:

Yes No Unsure

1. Exclusively in traditional working hours (9am to 5pm)
2. Evenings and weekends
3. Not on same day as other tests or clinic appointments
4. Based on your experience so far, would you recommend taking part in a research study with imaging to others? If no, please detail: Yes No Unsure
5. Is there anything else you would like to tell us about your experience of being invited to participate in trials with imaging?

**Thank you for taking the time to complete this questionnaire.**
